# Harmonized ^1^H NMR Workflow Enables Quantitative Betaine Determination from Nontargeted Metabolite Profiling Using Internal and External Standards

**DOI:** 10.1021/acs.analchem.5c05198

**Published:** 2026-05-29

**Authors:** Biagia Musio, Maria Trisolini, Rosa Ragone, Stefano Todisco, Antonino Rizzuti, Piero Mastrorilli, Mario Latronico, Nicola Intini, Annamaria Greco, Marica Antonicelli, Cristina Airoldi, Luca Antoniacomi, Luca Goldoni, Mihaela Balan-Porcăraşu, Francesca Benevelli, Davide Bertelli, Aurimas Bieliauskas, Luana Bontempo, Asma Bourafai-Aziez, Diego Brancaccio, Emanuela Callone, Angeles Canales Mayordomo, Enrico Caneva, Greta Petrella, Roberto Consonni, Iain J. Day, Catherine Deborde, Calin Deleanu, Giacomo Di Matteo, Cătălin Duduianu, John Edwards, Luca Fusaro, Sylvie Gehanne, Nicola Genna, Dessislava Gerginova, Roberto Gobetto, Gonzalo Hernandez, Nunzia Iaccarino, Pasquale Illiano, István Timári, Thomas Kuballa, Dirk W. Lachenmeier, Yavor Mitrev, Roland Molinie, Adele Mucci, Claudia Napoli, Alina Nicolescu, Valentina Petrelli, Luca Piemontese, Chiara Portesi, Antonio Randazzo, Teresa Recca, Algirdas Šačkus, Mirjam Schmidt, Svetlana Simova, Anatoly Petrovich Sobolev, Pavel Solovyev, Mattia Spano, Jan Teipel, Marina Veronesi, Vito Gallo

**Affiliations:** † Dipartimento di Ingegneria Civile, Ambientale, del Territorio, Edile e di Chimica (DICATECh), 124248 Politecnico di Bari, Via Orabona 4, I-70125 Bari, Italy; ‡ Innovative Solutions, Innovative Solutions S.r.l, Spin Off del Politecnico di Bari, Zona H 150/B, I-70015 Noci (BA), Italy; § Agenzia Regionale per la Prevenzione e la Protezione dell’Ambiente, 374338 ARPA Puglia, Corso Trieste 127, I-70126 Bari, Italy; ∥ Dipartimento di Biotecnologie e Bioscienze, Università of Milano-Bicocca, P.zza della Scienza 2, I-20126 Milano, Italy; ⊥ Comprehensive Substances characterization via advanced Spectroscopy (COSPECT), 9305 Università degli studi di Milano, Via Golgi 19, I-20133 Milano, Italy; # Analytical Chemistry Facility, 121451 Fondazione Istituto Italiano di Tecnologia (IIT), Via Morego 30, I-16163 Genoa, Italy; ∇ “Petru Poni” Institute of Macromolecular Chemistry, 41A Gr. Ghica Voda Alley, Iasi 700487, Romania; ○ Bruker Italia S.r.l., Viale V. Lancetti 43, I-20158 Milano, Italy; ◆ Dipartimento Scienze Della Vita, 9306 Università di Modena e Reggio Emilia, Via campi 103, I-41125 Modena, Italy; ¶ Institute of Synthetic Chemistry, 70309 Kaunas University of Technology, K. Baršausko Str. 59, LT-51423 Kaunas, Lithuania; †† Traceability Unit, 17954 Research and Innovation Center − Fondazione Edmund Mach (F.E.M), Via E. Mach 1, 38098 San Michele all’Adige (TN), Italy; ‡‡ EVEAR EXTRACTION, 48 Route de Gennes, LD Félines, CEDEX 4 F-49320 Angers France; §§ Dipartimento di Farmacia, 9307 Università di Napoli Federico II, Via D. Montesano 49, 80131 Napoli, Italy; ∥∥ Dipartimento di Ingegneria Industriale, Università di Trento, Via Sommarive 9, I-38123 Trento, Italy; ⊥⊥ Departamento Química Orgánica I, Facultad Ciencías Químicas, Universidad Complutense de Madrid, Avenida Complutense s/n., S-28040 Madrid, Spain; ## Dipartimento di Scienze e Tecnologie Chimiche, Università di Roma “Tor Vergata”, I-00133 Roma, Italy; ∇∇ Istituto di Scienze e Tecnologie Chimiche ″G. Natta″ (SCITEC), Consiglio Nazionale delle Ricerche (CNR), Via Corti 12, I-20133 Milan, Italy; ○○ 320944 JEOL UK Ltd, 4 Bankside, Long Hanborough, Oxfordshire OX29 8LJ, U.K.; ◆◆ MetaboHUB-Bordeaux, Centre INRAE de Nouvelle-Aquitaine Bordeaux, 71 Avenue E. Bourlaux, F-33140 Villenave d’Ornon, France; ¶¶ 200915 “C.D. Neniṭescu” Institute of Organic and Supramolecular Chemistry, 202-B Spl. Independenṭei, Bucharest RO-060023, Romania; ††† Dipartimento di Chimica e Tecnologie del Farmaco, 9311 Sapienza Università di Roma, Piazzale Aldo Moro 5, I-00185 Roma, Italia; ‡‡‡ Faculty of Chemical Engineering and Biotechnologies, 195061 National University for Science and Technology Politehnica Bucharest, Bucharest RO-011061, Romania; §§§ 377167 Process NMR Associates, LLC, 84 Patrick Lane, Suite 115, Poughkeepsie, New York 12603, United State; ∥∥∥ Chemistry Department, 54501 University of Namur, rue de Bruxelles 61, Namur 5000, Belgium; ⊥⊥⊥ 71514 Aptuit S.r.l. an Evotec company, Via Fleming 4, Verona 37135, Italy; ### Lab. Instruments S.r.l., SS172 Putignano-Alberobello Km 28 + 200, Castellana Grotte (BA) 70013, Italy; ∇∇∇ Institute of Organic Chemistry with Centre of Phytochemistry, 54525 Bulgarian Academy of Sciences, Akad. G. Bonchev bl. 9, Sofia 1113, Bulgaria; ○○○ Dipartimento di Chimica, 9314 Università di Torino, Via P. Giuria, 7, Torino I-10125, Italy; ◆◆◆ Laboratorio de Resonancia Magnética Nuclear, Departamento de Química Orgánica, Facultad de Química, 56724 Universidad de la República, Montevideo 11800, Uruguay; ¶¶¶ Department of Organic Chemistry, Faculty of Science and Technology, 37599 University of Debrecen, Egyetem tér 1, Debrecen H-4032, Hungary; †††† 208062 Chemisches und Veterinäruntersuchungsamt (CVUA) Karlsruhe, Weissenburger Strasse 3, Karlsruhe 76187, Germany; ‡‡‡‡ Transfrontalière BioEcoAgro, INRE 1158 Biologie des Plantes et Innovation (BIOPI), UPJV, 1; Rue des Louvels, Amiens 80037, France; §§§§ Dipartimento di Scienze Chimiche e Geologiche, Università di Modena e Reggio Emilia, Via G. Campi 103, 41125 Modena, Italy; ∥∥∥∥ FONDAZIONE ITS Istituto Tecnico Superiore Area “Nuove Tecnologie per il Made in Italy”, Settore produzioni agroalimentari; S.C. 138 C.da Marangi n.26, 70010 Locorotondo (Ba), Italy; ⊥⊥⊥⊥ 9295 University of Bari Aldo Moro, Dipartimento di Farmacia-Scienze del Farmaco, campus E. Quagliariello - Palazzo V. Tortorella, via E. Orabona 4, 70126 Bari, Italy; #### 83364 INRIM Istituto Nazionale di Ricerca Metrologica, Strada delle cacce 91, Torino 10135, Italy; ∇∇∇∇ Centro Grandi Strumenti, 428665 Università di Pavia, Via Bassi 21, Pavia 27100, Italy; ○○○○ 272865 Lower Saxony State Office for Consumer Protection and Food Safety (LAVES); Food and Veterinary Institute Braunschweig/Hannover, Dresdenstrasse 2, Braunschweig 38124, Germany; ◆◆◆◆ 366605 Istituto per i Sistemi Biologici; Consiglio Nazionale delle Ricerche CNR; via Salaria km 29.300, Monterotondo (RM) 00015, Italy; ¶¶¶¶ Structural Biophysics Facility, Fondazione Istituto Italiano di Tecnologia (IIT), Via Morego 30, I-16163 Genoa, Italy

## Abstract

Nontargeted metabolite profiling prioritizes robust comparisons of the analytical outcomes rather than absolute concentration measurement. In this work, it is shown that a harmonized 1D ^1^H NMR workflow, originally adopted for nontargeted NMR analysis, can also support reliable quantitative determination of betaine when spectra acquired under profiling-oriented conditions, nonideal for quantification, are anchored to gravimetrically traceable standards and corrected by a suitable factor accounting for bias in absolute concentration estimates. This study presents the results of an interlaboratory comparison designed to investigate the main factors affecting the accuracy and reproducibility of nontargeted ^1^H NMR data when different spectrometers and operators are involved. The case study focused on the determination of betaine in aqueous extracts of durum wheat (cvs. *Marco Aurelio* and *Iride*) and the corresponding pasta products. A common set of samples was analyzed using a harmonized acquisition protocol across 50 spectrometers operating at magnetic field strengths ranging from 80 to 700 MHz. Two data-processing strategies were compared: operator-dependent processing (multiple operators using different software packages) and centralized processing (single operator) performed with five different software platforms. Quantification was carried out by both an internal standard method, using 3-(trimethylsilyl)-2,2,3,3-tetradeutero-propionic acid, sodium salt (TSP-*d*_4_) as a reference, and an external standard method, employing TSP-*d*_4_, dimethyl sulfone (DMSO_2_), and betaine as references. The results demonstrated that the largest source of variability lies in operator-dependent data-processing choices rather than instrumental characteristics. TSP-*d*_4_ systematically overestimated the betaine concentration and introduced additional variability. By contrast, DMSO_2_ and betaine provided accurate and highly precise quantification with Horwitz ratios consistently below unity, indicating reproducibility superior to generic interlaboratory expectations. Internal standard method also achieved reproducibility within the accepted 0.5–2.0 HorRat range. Overall, this work shows that spectra acquired for nontargeted metabolite profiling can support quantitative determination of betaine, and potentially of other selected metabolites, provided that the same acquisition and processing protocol is maintained and that appropriate gravimetrically traceable calibration is applied.

## Graphical abstract

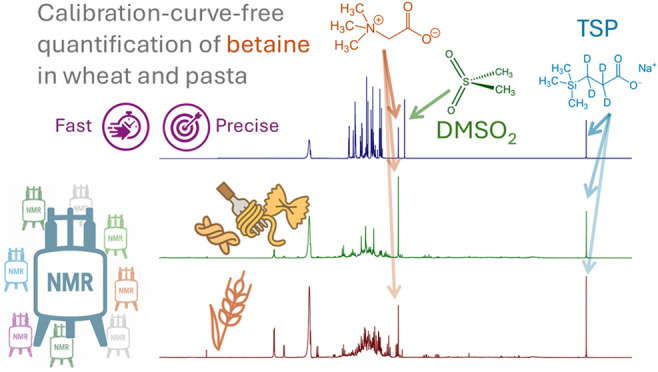

## Introduction

Quantitative analysis using nuclear magnetic resonance (NMR) is becoming one of the pillars of contemporary analytical chemistry, mainly due to its unrivaled ability to provide information about molecular structures while offering quantitative information. The versatility of NMR is evident in its wide range of applications, spanning from organic and materials chemistry to pharmaceutical research and food sciences.^1–5^ However, the accurate exploitation of NMR for quantitative purposes requires careful consideration of relaxation phenomena, which play a crucial role in both signal generation and data interpretation. In particular, ensuring adequate nuclear relaxation between successive scans is essential to avoid systematic biases in the quantitative results. Proper management of pulse sequences and relaxation delays is not merely a technical detail but a decisive factor that governs the reliability of NMR as a quantitative tool. When these parameters are carefully optimized, NMR offers the unique advantage of providing accurate and precise results typical of quantitative analysis. In this context, NMR is recognized as a primary mass ratio method, it is metrologically equivalent to a mass balance methodology for purity and content determination, and it is referred to as quantitative NMR (qNMR).^6–10^

However, in routine metabolomics, NMR measurements are often exploited with a nontargeted approach, i.e., as a high-throughput platform for metabolite profiling where a single reference compound provides approximate scaling across many metabolites without compound-specific calibration curves.^3–5,11^ In practice, profiling-oriented NMR workflows are often optimized for broad metabolite coverage and intersample comparability rather than for strict quantitative performance for every individual analyte. Under such nonideal acquisition conditions, including repetition times shorter than those typically required for full longitudinal relaxation, absolute concentrations may be biased even when relative trends remain reproducible.

The two distinct modes of use highlight the flexibility of NMR and underscore the importance of aligning its application with an analytical objective. The present study aims to demonstrate that an NMR workflow originally developed for nontargeted profiling, under appropriate conditions, can yield analyte concentrations with uncertainties and metrological rigor comparable to those of dedicated quantitative approaches. The work focuses on assessing potential biases associated with relative quantification using NMR spectra specifically acquired for nontargeted analyses and in the absence of a calibration curve. In particular, it evaluates three key factors influencing measurement precision under these conditions: (a) the statistical distribution of the spectroscopic data produced by 50 different spectrometers; (b) potential biases arising from the spectral processing procedure; and (c) the reproducibility of the analytical results obtained by employing both internal and external standards. These attributes are essential for the reliable application of nontargeted NMR across distributed analytical settings.

Betaine quantification was selected as a case study because betaine is a metabolite of agronomic and nutritional relevance.^12–15^ Its monitoring is crucial for breeding strategies and crop quality assessment. Several qNMR methods have already been reported for quantitative analysis of betaine and other bioactive constituents in complex mixtures, including herbal products and nutraceutical preparations.^16–18^ These studies are based on dedicated qNMR acquisition schemes optimized *a priori* for quantitative purposes. The objective of this study is not to re-establish the quantitative validity of NMR for betaine but to evaluate profiling-oriented NMR experiments as an efficient analytical strategy. This approach avoids the need to redesign acquisition protocols for each individual analyte and, under appropriate processing conditions, may be extended to other molecules in the same sample.

To test this hypothesis, an interlaboratory comparison was conducted in 2022 involving 50 NMR facilities worldwide. The study was designed to validate a nontargeted NMR protocol for the determination of betaine in aqueous extracts of wheat and pasta. Betaine provides a relevant case study to assess whether nontargeted NMR data can support rigorous *a posteriori* quantification when the protocol is anchored to suitable gravimetrically traceable standards and an appropriate correction strategy. An internal standard method using TSP-*d*_4_ was evaluated and compared with the external calibration methods. The external calibration approaches employed TSP-*d*_4_, dimethyl sulfone (DMSO_2_), and betaine as external standards. The effects of operator variability and software selection on data quality were also evaluated, and corresponding precision benchmarks were established.

## Material and Methods

### Materials

3-(Trimethylsilyl)-2,2,3,3-tetradeutero-propionic acid, sodium salt (TSP-*d*_4_, CAS N. 24493–21–8, 99%D, Batch 6712, Armar Chemicals, Döttingen, Switzerland), sodium azide (NaN_3_, CAS N. 26628–22–8; ≥99.5%, Sigma-Aldrich, Milan, Italy), and deuterium oxide (D_2_O, CAS. N. 151882–100G; 99.9%D, Sigma-Aldrich, Milan, Italy), sodium oxalate (Na_2_C_2_O_4_, CAS N. 62–76–0, ≥99.5%, Sigma-Aldrich, Milan, Italy), hydrochloric acid (HCl, CAS N. 7647–01–0, 37%, Sigma-Aldrich, Milan, Italy), and methanol-*d*_4_ (CD_3_OD, CAS. N. 811–98–3, 99%D, Lot: MKCN9119; Sigma-Aldrich, Milan, Italy), D (−)-fructose (fructose, CAS: 57–48–7, 99%, Lot: A0282904, Acros Organics B.V.B.A., Geel, Belgium), certified powder of betaine (CAS: 107–43–7; Pharmaceutical Secondary Standard, 99.99%, Lot No.: LRAC 5297, EXP: Feb/24, Merck, Buchs, Switzerland), and certified powder of dimethyl sulfone (DMSO_2_, CAS: 67–71–0, qNMR TraceCERT, 99.96%, Lot No.: BCCH 9571, EXP: Sept/26, Merck, Buchs, Switzerland) were used for sample preparation. NMR tubes (Wilmad WG-1000–7) were purchased from Sigma-Aldrich, Milan, Italy. Poly­(vinylidene fluoride) (PVDF) centrifuge filters (mod. F2519–5, pore size of 0.2 μm, Volume 25 mL) were purchased from Thermo Fischer Scientific, Monza, Italy.

Wheat samples were provided by Azienda Agricola Denora (Altamura, Bari, Italy) while pasta samples were provided by Casa Prencipe (Monte S. Angelo, Foggia, Italy) in the framework of the project IPERDURUM (www.iperdurum.it).

### Aqueous Extracts of Wheat and Pasta (Bulk Solution)

For each cultivar (*Triticum durum* *cv. Marco Aurelio* and *T. durum* *cv. Iride*), 50 g of whole wheat (or pasta, type Caserecce) were ground using a blender for 1.0 min and sieved using a laboratory sieve having a size of 0.5 mm. The bulk solution of the extracts was prepared by treating 6.0 g of the powder with 60 mL of a solution containing oxalate buffer ([HC_2_O_4_^–^/C_2_O_4_^2–^] = 0.25 M, pH = 4.2) and sodium azide (NaN_3_ = 2.5 × 10^–3^ M). The resulting mixture was submitted to sonication through an ultrasonic bath at 40 kHz at 60 °C for 10 min, then to Vortex mixing at 2500 rpm for 5 min, and finally to centrifugation at 4000 rpm for 10 min. Supernatant was collected into a bottle and pasteurized at 90 °C for 10 min. After pasteurization, the solution was further centrifuged at 4000 rpm for 5 min in poly­(vinylidene fluoride) centrifuge filters (PVDF, pore size of 0.2 μm). Each filtered solution was stored overnight at 4 °C.

### Solution Containing the Reference Compounds

The aqueous solution containing the reference compounds was prepared by dissolving 19.355 mg of TSP-*d*_4_, 19.281 mg of betaine, 19.719 mg of DMSO_2_, and 1.00011 g of D (−)-fructose in 100 mL of the buffer solution ([HC_2_O_4_^–^/C_2_O_4_^2–^] = 0.25 M, pH = 4.2; NaN_3_ = 2.5 × 10^–3^ M).

The resulting mixture was used to fill the QR tube.

### Sample Preparation

NMR tubes were filled in, using an automated system for liquid handling (SamplePro, Bruker BioSpin GmbH, Rheinstetten, Germany), by adding 630 μL of the obtained extracts (or the solution containing the reference compounds) and 70 μL of either D_2_O or a solution of TSP-*d*_4_ in D_2_O (0.20%, w/w). Additionally, NMR tubes containing 630 μL of methanol-*d*_4_ were prepared and used for temperature calibration at 298.0 ± 0.1 K according to the procedure reported by Findeisen et al.^19^ All NMR tubes were flame-sealed before delivery.

A total of 8 NMR tubes were provided for each participant. As detailed in Table S1, three tubes contained buffered aqueous extracts of pasta (A, B, and C), three tubes contained buffered aqueous extracts of wheat (D, E and F), one tube (QR) contained aqueous solutions of reference compounds, and one tube (T) contained methanol-*d*_4_.

Stability tests were carried out following the procedure reported in the Supporting Information.

Homogeneity test was carried out on the entire batch made up of 350 NMR tubes [7 NMR tubes (A-F and QR) multiplied by 50 participants] before delivery to the participants, according to ISO/IEC 17043:2010.^20^ The 350 NMR tubes were submitted to 1D ^1^H NOESY experiments preceded by a selective presaturation step by a Bruker Avance I 400 spectrometer equipped with a 5 mm inverse probe.

### 1D ^1^H NOESY Experiments Carried Out in the ILC

According to the guidelines for the participants,^21^ tubes A-F and QR were used as test samples. The spectrometers were from different manufacturers (Agilent, Bruker, and JEOL) and differed in magnetic field, production year, and hardware configurations. The magnetic field strength of 50 spectrometers could be subdivided into: 80 MHz (*n* = 3); 300 MHz (*n* = 1); 400 MHz (*n* = 20); 500 MHz (*n* = 4); 600 MHz (*n* = 17); 700 MHz (*n* = 5). The data acquisition setup (Table S2) was set to ensure a spectral window of 15 ppm, an acquisition time of 4.267 s, and a digital resolution of 0.1172 Hz for all magnetic fields.

In the following, other parameters are indicated according to the vendor language. For Agilent spectrometers, pulse program: NOESY; time domain (np) as indicated in Table S2; spectral width (sw) as indicated in Table S2; transmitter offset (tof): ca. 4.70 ppm (chemical shift value of the residual water signal); steady state (ss): 8; number of transient (nt): 64; mixing time (mixN): 0.01 s; recycle delay (d1): 6 s; no sspul (sspul = “*n*”); no ZQ filter (Gzqfil = “*n*”); no homo spoil during mixing time (gt1 = 0, gzlvl1 = 0 and gstab = 0. For Bruker spectrometers, pulse program: noesypr1d; time domain (TD) as indicated in Table S2; spectral width (SW) as indicated in Table S2; transmitter offset (O1P): ca. 4.70 ppm (chemical shift value of the residual water signal); dummy scans (ds): 8; number of scans (ns): 64; mixing time (d8): 0.01 s; recycle delay (d1): 6 s. For JEOLspectrometers, pulse program: noesy_abs; *y*_points: 1; time domain (*x*_points) as indicated in Table S2; spectral width (*x*_sweep) as indicated in Table S2; transmitter offset (*x*_offset): ca. 4.70 ppm (chemical shift value of the residual water signal); steady state (*x*_prescans): 8; number of transients (scans): 64; mixing time (mix_time): 0.01 s; recycle delay (relaxation_delay): 6 s.

### NMR Data Processing

Each participant acquired 35 NMR spectra (tubes A-F and QR, 5 repetitions per tube) and processed the raw data (free induction decays) by applying Fourier transform using an exponential multiplication function with a line broadening of 0.1 Hz. Phase and baseline corrections and signal integration were performed according to individual choices of the users by software packages available in their own laboratory. The spectra were referenced to the betaine peak (singlet) at 3.25 ppm. Totally, this resulted in 1750 NMR spectra, deriving from 35 data sets generated by 50 spectrometers. In the following, the results derived from the collective elaboration of the NMR data provided by the participants will be referred to as multioperator results.

Subsequently, the same raw data (1750 data sets) were processed by a single operator using TOPSPIN 3.0 (Bruker BioSpin GmbH, Rheinstetten, Germany). Fourier transform was performed using an exponential multiplication function with a line broadening of 0.1 Hz. The phase was manually corrected, and the baseline was fitted to a polynomial line of degree 1. The spectra were referenced to the betaine peak (singlet) at 3.25 ppm. Then, betaine, DMSO_2_, and TSP-*d*_4_ peaks were submitted to integration by using five different software applications: TOPSPIN 3.0, AMIX 3.9.11 (Bruker BioSpin GmbH, Rheinstetten, Germany), MestreNova 14.3.3 (Mestrelab Research, Santiago de Compostela, Spain), ACD/Laboratories 2015 (NMR Workbook Suite, Advanced Chemistry Development, Inc., Toronto, Ontario, Canada), and JASON dev-Version 4.2.8147 (JEOL JASON, 4 Bankside, Long Hanborough, Witney, UK). In the following, the results derived from NMR data elaborated by the selected single user will be termed single operator results.

### Statistical Treatment of the NMR Data

Data treatment regarding tubes A, B, E, and F was as follows: the betaine signal integral was scaled to the TSP-*d*_4_ signal integral, and the corresponding *I*_betaine_/*I*_TSP‑*d*4_ values were uploaded by each participant on the Website http://nmr.mxcs.it/index.php, specifically designed and validated for data elaboration in agreement with internationally accepted requirements.^20^ All statistical analyses and calculations^22–24^ were performed automatically by a dedicated routine implemented on the Website, ensuring a standardized and operator-independent data treatment across participants. *I*_betaine_/*I*_TSP‑*d*4_ values were uploaded with at least 8 decimal places. The workflow of the dedicated routine consisted of intralaboratory (Step 1) and interlaboratory (Step 2) elaborations.

*Step 1*. The five repetitions (*I*_betaine_/*I*_TSP‑*d*4_ values provided for each tube) produced by each spectrometer were submitted to Huber, Dixon, and Grubbs tests for the identification of outliers. Values simultaneously identified as outliers by all three outlier tests were removed from the data set and were not considered in successive calculations. After removing outliers, the remaining *I*_betaine_/*I*_TSP‑*d*4_ values were used to determine the average value and the corresponding standard deviation, which was considered as intralaboratory uncertainty of the method.

*Step 2*. The refined data obtained according to the workflow reported in Step 1 were submitted to data elaboration for the determination of the assigned interlaboratory *I*_betaine_/*I*_TSP‑*d*4_ values. According to Horwitz,^22^ the refined data were submitted to the Cochran test with the aim to identifying and removing the data sets characterized by a relative standard deviation which resulted unusually large compared with the others. Thus, outliers in dispersion were not considered for successive calculations. The average *I*_betaine_/*I*_TSP‑*d*4_ values from the remaining data sets were submitted to the Huber test with the aim of finding a reliable average and spread by reducing the influence of extreme results. Finally, all the average *I*_betaine_/*I*_TSP‑*d*4_ values that successfully passed both Cochran and Huber tests were submitted to Shapiro-Wilk and D’Agostino tests to ascertain the normal distribution of the population and were used to calculate the consensus *I*_betaine_/*I*_TSP‑*d*4_ value (interlaboratory average),^23,24^ and the corresponding relative standard deviation (RSD%).

## Results and Discussion

### Introduction and Calculation of the Protocol-Dependent Correction Factor α under Nonideal Conditions

Under ideal quantitative NMR (qNMR) analytical conditions, including the ideality of the solution and an optimal array of acquisition conditions (typically defined by full relaxation, e.g., repetition time ≥5 × *T*_1_, appropriate flip angle, and absence of saturation effects), the peak(s) integral is directly proportional to the number of resonant nuclei contributing to that signal according to

1 I=k·n

where *I* is the integral of the peak(s) generated by the number of moles (*n*) of nuclei and *k* is a spectrometer constant valid for all peaks within the same NMR spectrum. In this context, internal standard methods rely on the fundamental proportionality between the peak integral and number of moles, allowing the ratio of two integrals within the same spectrum to reflect the ratio of the corresponding nuclei mole numbers. Importantly, since qNMR is recognized as a primary method of measurement, the equivalence between the mole ratio determined by qNMR and that obtained by gravimetric preparation can be established under properly controlled experimental conditions. In other words, under ideal analytical conditions, the mole ratio derived by qNMR is metrologically consistent with that defined by gravimetric measurements. Thus, considering an internal standard qNMR method^25^ as a representative example, the ratio between the analyte signal and the reference signal can be written as

2 $${\left(\frac{I_{\mathrm{anl}}}{I_{\mathrm{ref}}}\right)}_{\mathrm{qNMR}}={\left(\frac{n_{H,\mathrm{anl}}}{n_{H,\mathrm{ref}}}\right)}_{\mathrm{qNMR}}={\left(\frac{n_{H,\mathrm{anl}}}{n_{H,\mathrm{ref}}}\right)}_{\mathrm{gravimetric}}$$

where *I*_anl_ and *I*_ref_ represent the peak(s) integrals obtained under ideal acquisition conditions, *n*_H,anl_ and *n*_H,ref_ are the moles of the hydrogen atoms generating the signals, which are consistent with the corresponding gravimetric amounts. This equivalence underpins the traceability of qNMR results to International System of Units (SI) and constitutes the foundation of its use in purity assessment and reference standard characterization. In fact, making explicit the moles of hydrogen atoms generating the signals, eq 2 can be rewritten to account for concentrations as follows

3 $${\left(\frac{I_{\mathrm{anl}}}{I_{\mathrm{ref}}}\right)}_{\mathrm{qNMR}}={\left(\frac{n_{H,\mathrm{anl}}}{n_{H,\mathrm{ref}}}\right)}_{\mathrm{gravimetric}}=\frac{N_{H,\mathrm{anl}}}{N_{H,\mathrm{ref}}}\cdot {\left(\frac{n_{\mathrm{anl}}}{n_{\mathrm{ref}}}\right)}_{\mathrm{gravimetric}}=\frac{N_{H,\mathrm{anl}}}{N_{H,\mathrm{ref}}}\cdot {\left(\frac{\left[\mathrm{anl}\right]}{\left[\mathrm{ref}\right]}\right)}_{\mathrm{gravimetric}}$$

where *N*_H,anl_ and *N*_H,ref_ are the number of chemically and magnetically equivalent hydrogen atoms contained in the molecule and generating the signals, *n*_anl_, *n*_ref_, [anl], and [ref] are the numbers of moles and the molar concentrations of the analyte and reference molecules, respectively. This proportionality constitutes the fundamental principle of the internal standard qNMR method, allowing for the calculation of the analyte concentration by rearranging the first and the last terms as follows

4 $${\left[\mathrm{anl}\right]}_{\mathrm{qNMR}}={\left[\mathrm{anl}\right]}_{\mathrm{gravimetric}}={\left(\frac{I_{\mathrm{anl}}}{I_{\mathrm{ref}}}\right)}_{\mathrm{qNMR}}\cdot \frac{N_{H,\mathrm{ref}}}{N_{H,\mathrm{anl}}}\cdot {\left[\mathrm{ref}\right]}_{\mathrm{gravimetric}}$$

Under nonideal conditions, as for acquisitions carried out with repetition times shorter than 5 × *T*_1_, (nontargeted NMR conditions, ntNMR) the consistency between the mole ratio derived spectroscopically and that gravimetrically determined is lost. Consequently, eq 2 should be rewritten as

5 $${\left(\frac{I_{\mathrm{anl}}}{I_{\mathrm{ref}}}\right)}_{\mathrm{ntNMR}}={{\left(\frac{n_{H,\mathrm{anl}}}{n_{H,\mathrm{ref}}}\right)}_{\mathrm{ntNMR}}=\left(\frac{n_{H,\mathrm{anl}}}{n_{H,\mathrm{ref}}}\right)}_{\mathrm{spectroscopic}}$$

The discrepancy between the gravimetric mole ratio (eq 2) and the spectroscopic mole ratio (eq 5) arises because, under nonideal conditions, the NMR integral is more appropriately expressed as

6 $$I_{\mathrm{ntNMR}}=k\cdot n\cdot f$$

where *k* and *n* are the same physical quantities as in eq 1, *I*_ntNMR_ is the integral of the signal generated under nonideal conditions, and *f* represents a recovery factor associated with incomplete longitudinal relaxation. Factor *f* is nucleus-specific and may differ between analyte and reference signals. Thus, under nontargeted NMR conditions, *f* should be included in the combination of the eqs 3, 5, and 6 to give

7 $${\left(\frac{I_{\mathrm{anl}}}{I_{\mathrm{ref}}}\right)}_{\mathrm{ntNMR}}={\left(\frac{n_{H,\mathrm{anl}}}{n_{H,\mathrm{ref}}}\right)}_{\mathrm{spectroscopic}}={\left(\frac{n_{H,\mathrm{anl}}}{n_{H,\mathrm{ref}}}\right)}_{\mathrm{gravimetric}}\cdot \frac{f_{H,\mathrm{anl}}}{f_{H,\mathrm{ref}}}=\frac{N_{H,\mathrm{anl}}}{N_{H,\mathrm{ref}}}\cdot {\left(\frac{n_{\mathrm{anl}}}{n_{\mathrm{ref}}}\right)}_{\mathrm{gravimetric}}\cdot \frac{f_{H,\mathrm{anl}}}{f_{H,\mathrm{ref}}}$$

From eq 7, it can be deduced that the mole ratio metrologically consistent with the gravimetric measurement is related to the mole ratio spectroscopically determined under nonideal acquisition conditions through the $\frac{f_{H,\mathrm{anl}}}{f_{H,\mathrm{ref}}}$ ratio accounting for incomplete longitudinal relaxation of the nuclei.

Defining α as the reciprocal of $\frac{f_{H,\mathrm{anl}}}{f_{H,\mathrm{ref}}}$ , it follows that

8 $$\alpha =\frac{f_{H,\mathrm{ref}}}{f_{H,\mathrm{anl}}}=\frac{{\left(\frac{n_{H,\mathrm{anl}}}{n_{H,\mathrm{ref}}}\right)}_{\mathrm{gravimetric}}}{{\left(\frac{n_{H,\mathrm{anl}}}{n_{H,\mathrm{ref}}}\right)}_{\mathrm{spectroscopic}}}=\frac{{\left(\frac{I_{\mathrm{anl}}}{I_{\mathrm{ref}}}\right)}_{\mathrm{qNMR}}}{{\left(\frac{I_{\mathrm{anl}}}{I_{\mathrm{ref}}}\right)}_{\mathrm{ntNMR}}}$$

Accordingly, α should be viewed as a protocol-dependent correction factor that compensates for bias arising under nonideal acquisition conditions (α ≠ 1) and restores metrological consistency (α = 1) between spectroscopic and gravimetrically traceable mole ratios. Correction factors like α are not unusual in quantitative NMR.^26^ In routine application, α can be exploited to calculate the analyte concentration [anl] with metrological consistency by combining eqs 4 and 8 as follows

9 $$\left[\mathrm{anl}\right]=\alpha \cdot {\left(\frac{I_{\mathrm{anl}}}{I_{\mathrm{ref}}}\right)}_{\mathrm{ntNMR}}\cdot \frac{N_{H,\mathrm{ref}}}{N_{H,\mathrm{anl}}}\cdot {\left[\mathrm{ref}\right]}_{\left(\mathrm{gravimetric}\right)}$$

In the present study, α was evaluated by NMR experiments conducted on a model mixture (QR solution) under conditions far from ideality. In fact, the repetition time (the sum of acquisition time and recycle delay) was kept approximately at 10 s against 21 s required for a complete recovery of the magnetization vector, in agreement with the highest *T*_1_ value found for DMSO_2_ (4.17 s) in Tube QR (Supporting Information). These conditions were chosen with the aim of shortening the total experiment time and avoiding possible hardware drawbacks deriving from a long-lasting presaturation step, particularly when investigating water-soluble metabolites. The study was conducted by analyzing the QR solution and comparing the betaine/TSP-*d*_4_, betaine/DMSO_2_, and DMSO_2_/TSP-*d*_4_ mole ratios determined by both nontargeted NMR (as ratios between the integrals of the methyl hydrogen signals labeled as betaine, DMSO_2_, and TSP-*d*_4_ in Figure 1) and gravimetric procedure (as ratios between the moles of the methyl hydrogen atoms calculated considering the masses of the analytes introduced into the sample).

**Figure 1:**
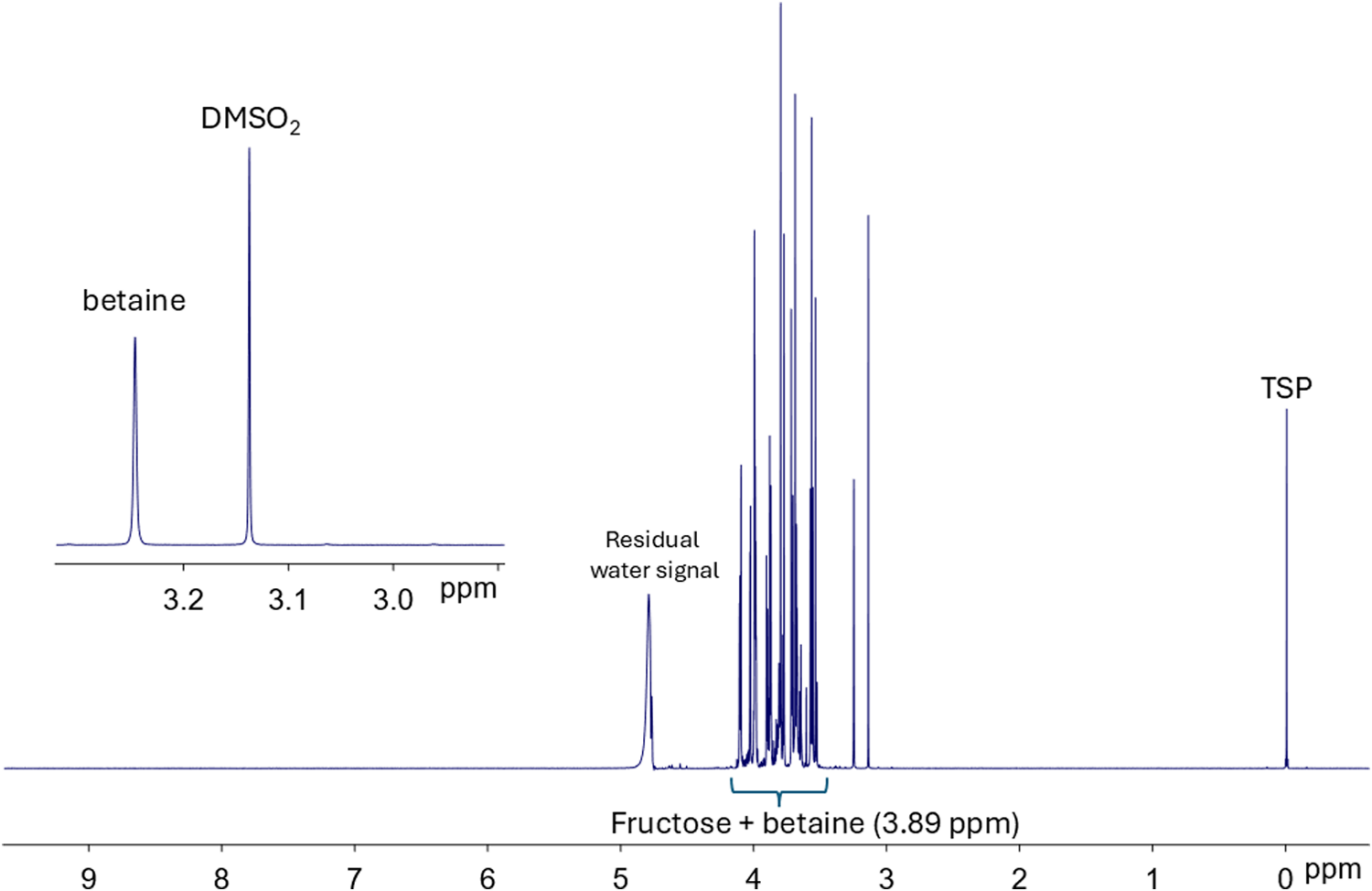
Typical 1D ^1^H NOESY spectrum of a model mixture (Tube QR, 400 MHz, 298 K).

In Table 1, the spectroscopic mole ratios were calculated by a single operator considering the appropriate peak integrals in the 250 spectra of the reference mixture (QR tube), whereas gravimetric results were calculated by the host laboratory using information from the amounts of the introduced reference materials.

**Table 1:** Spectroscopic and Gravimetric Mole Ratios of the Reference Compounds in Tube QR

	betaine/TSP-*d*_4_	betaine/DMSO_2_	DMSO_2_/TSP-*d*_4_
${\left(\frac{n_{H,\mathrm{anl}}}{n_{H,\mathrm{ref}}}\right)}_{\mathrm{spectroscopic}}$	1.652	1.193	1.386
RSD%_Spectroscopic_	4.32	4.00	4.23
${\left(\frac{n_{H,\mathrm{anl}}}{n_{H,\mathrm{ref}}}\right)}_{\mathrm{gravimetric}}$ ^a^	1.466	1.179	1.243
RSD%_gravimetric_	0.016	0.009	0.020
difference_(spectroscopic‑gravimetric)_ ^b^	0.186	0.014	0.143
α	0.887	0.988	0.897

a mole ratios were calculated according to eq 3, considering *N*_H,betaine_ = 9, *N*_H,DMSO_2__ = 6 and *N*_H,TSP‑*d*_4__ = 9.

b calculated as ${\left(\frac{n_{H,\mathrm{anl}}}{n_{H,\mathrm{ref}}}\right)}_{\mathrm{spectroscopic}}-{\left(\frac{n_{H,\mathrm{anl}}}{n_{H,\mathrm{ref}}}\right)}_{\mathrm{gravimetric}}$

In the cases of spectroscopic mole ratios, the RSD% values were comparable, varying between 4.00 and 4.32, with the slightly higher values observed in calculations involving TSP-*d*_4_. It is noteworthy that each RSD% value reported in Table 1 was calculated using the full set of 250 measurements without exclusion from potential outliers. Thus, the reported relative standard deviations represent a conservative estimate obtained under stringent statistical conditions yet remain within commonly accepted precision limits.

The systematic differences observed between spectroscopic and gravimetric determinations for the betaine/TSP-*d*_4_ and DMSO_2_/TSP-*d*_4_ ratios with respect to the betaine/DMSO_2_ ratios indicate an apparent underestimation of TSP-*d*_4_ mole amounts under the adopted experimental conditions. Such differences can be appreciated also in terms of the α values, going from 0.988 (for betaine/DMSO_2_) to 0.887 and 0.897 (for betaine/TSP-*d*_4_ and DMSO_2_/TSP-*d*_4_, respectively).

Based on the experimentally determined *T*_1_ values for TSP-*d*_4_ (3.18 s), DMSO_2_ (4.17 s), and betaine (2.05 s) at a magnetic field strength of 400 MHz, under the acquisition conditions adopted in this study (repetition time of 10 s), the greatest deviation from full relaxation would theoretically be expected for DMSO_2_, given its longer *T*_1_. Accordingly, a relative underestimation of the DMSO_2_ intensity would be anticipated, whereas TSP-*d*_4_ and betaine should be less affected. The experimental observations, therefore, suggest that relaxation effects alone do not fully explain the bias associated with TSP-*d*_4_. Additional factors, potentially including matrix interactions, intermolecular association, or microenvironmental effects, may contribute to the systematic attenuation of the TSP-*d*_4_ signal. While a detailed mechanistic investigation is beyond the scope of the present study, similar discrepancies involving TSP-*d*_4_ have been reported for other matrices.^27–29^ Nevertheless, these observations raise an intriguing question as to whether TSP-*d*_4_, with its availability, ease of handling, and long-standing tradition, can still serve as a reliable quantitative reference. This point is explored in the following sections, where TSP-*d*_4_ has been investigated as a reference molecule.

### Quantification of Betaine in Aqueous Extracts of Wheat and Pasta by Using TSP-*d*_4_ as an Internal Standard

Following the evaluation of analyte responses in the model mixture, attention was directed to the calculation of the betaine/TSP-*d*_4_ mole ratio in complex mixtures under the same ntNMR acquisition conditions (Figure 2) with the final aim of determining the betaine concentration.

**Figure 2:**
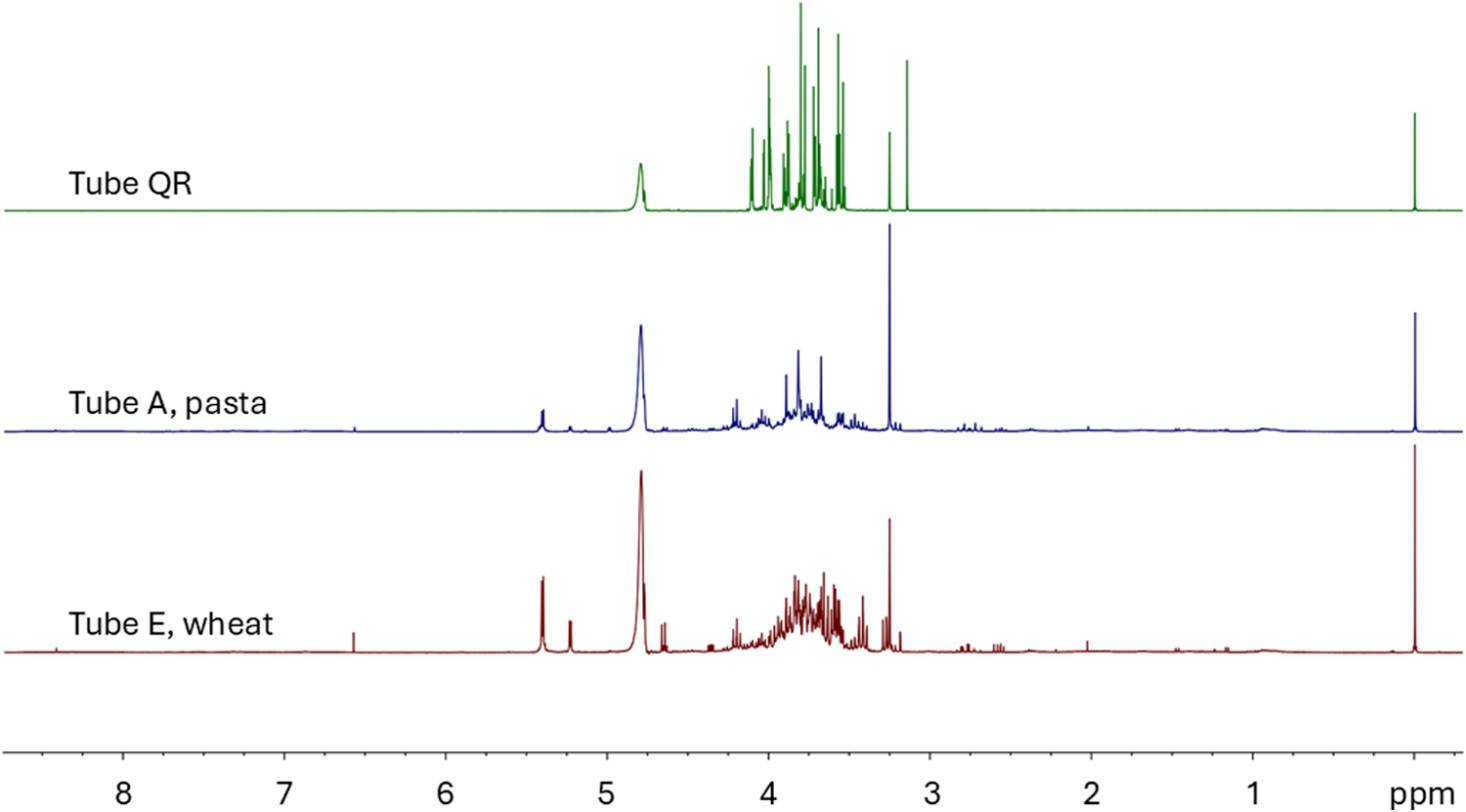
Typical 1D ^1^H NOESY spectra of the model mixture, aqueous extract of pasta, and aqueous extract of wheat (400 MHz, 298 K).

The reported consensus values of *I*_bet_/*I*_TSP‑*d*4_ in the aqueous extracts of pasta (tubes A and B, Table S1) and of wheat (tubes E and F, Table S1) were calculated according to the workflows described as Steps 1 and 2 (Table 1). The number of the *I*_bet_/*I*_TSP‑*d*4_ values remaining after the outlier removal is reported in Table 2 as the number of spectrometers participating in the consensus value. A critical component of this study was the comparison of two data-processing strategies applied to identical raw NMR data sets. In the multioperator protocol, each of the 50 participants independently processed their spectra using software of their choice. In contrast, the single-operator protocol involved centralized reprocessing of all FIDs by one operator using multiple software platforms (TOPSPIN, ACD/Laboratories, AMIX, MestreNova, and JASON), thereby ensuring consistent processing criteria across data sets.

**Table 2:** *I*_bet_/*I*_TSP‑d_4__ Determined for Aqueous Extracts of Pasta and Wheat

	multioperator results	single operator results				
	variable software applications	TOPSPIN	ACD-laboratories	AMIX	MestreNova	JASON
**Tube A Pasta (cv.** ***Iride*** **)**						
*I*_bet_/*I*_TSP‑*d*_4__	0.96	1.04	1.04	1.04	1.04	1.07
RSD%	5.77	10.09	10.09	10.10	10.11	10.89
Number of spectrometers participating in the consensus value	25	38	38	38	38	39
Outliers by Cochran test	18	7	7	7	7	6
Outlier by Huber test	7	5	5	5	5	5
**Tube B Pasta (cv.** ***Marco Aurelio*** **)**						
*I*_Bet_/*I*_TSP‑*d*_4__	1.67	1.77	1.77	1.77	1.77	1.82
RSD%	4.42	6.26	6.25	6.26	6.27	6.34
Number of spectrometers participating in the consensus value	21	36	36	36	36	36
Outliers by Cochran test	22	11	12	11	11	9
Outlier by Huber test	7	3	2	3	3	5
**Tube E Wheat (cv.** ***Marco Aurelio*** **)**						
*I*_bet_/*I*_TSP‑*d*_4__	2.50	2.63	2.64	2.63	2.64	2.77
RSD%	4.09	5.08	5.08	4.88	5.13	4.37
Number of spectrometers participating in the consensus value	28	43	47	43	47	42
Outliers by Cochran test	20	7	3	7	3	8
Outlier by Huber test	2	0	0	0	0	0
**Tube F Wheat (cv.** ***Iride*** **)**						
*I*_bet_/*I*_TSP‑*d*_4__	1.96	2.07	2.06	2.07	2.07	2.16
RSD%	4.27	5.43	5.43	5.43	5.44	5.27
Number of spectrometers participating in the consensus value	33	44	45	44	44	42
Outliers by Cochran test	14	6	5	6	6	8
Outlier by Huber test	3	0	0	0	0	0

The entries in Table 2 labeled “Number of spectrometers participating in the consensus value”, “Outliers by Cochran test”, and “Outliers by Huber test” were the direct output of the automated statistical treatment described in Steps 1 and 2 for NMR spectra acquired under nonideal quantification conditions. Briefly, repetitions were first evaluated at the intralaboratory level, after which refined data sets were subjected to Cochran’s test to identify unusually large intralaboratory dispersion. Subsequently, Huber’s robust procedure was applied to exclude extreme laboratory means before calculating the consensus *I*_bet_/*I*_TSP‑d4_ value and its associated RSD%.

The comparison between multioperator processing (each of the 50 participants processing their own spectra) and single-operator processing (one operator reprocessing all FIDs) indicates that, when processing was decentralized, a larger number of data sets were flagged as outliers, especially by Cochran’s test. It can be deduced that, in agreement with literature data,^30,31^ operator-dependent choices in phasing, baseline correction, and integration cause the increase of the intralaboratory variability. Conversely, using identical raw data, centralized processing by a single operator applying consistent criteria markedly reduced both dispersion outliers (Cochran) and extreme results (Huber). Therefore, the number of spectrometers retained for the consensus value calculation increased by more than 20% across the samples. It should also be noted that, in interlaboratory studies, the RSD% depends not only on analytical performance but also on the number and heterogeneity of the laboratories retained after outlier removal. Consequently, incorporating a larger set of spectrometers naturally increases interlaboratory RSD%, even though intralaboratory precision is improved by single-operator processing. Minor differences observed among the software applications are not substantial. While they can partly be attributed to the intrinsic characteristics of the respective algorithms, they are more likely the result of the specific rules and automation routines implemented by the operator within each platform. These operator-dependent choices, which influence how baseline correction, phasing, and integration are executed in practice, contribute to subtle variations that, however, remain negligible in their impact. Given the marginal extent of these differences, a detailed discussion was deemed to be unnecessary. The RSD% values reported in Table 2 seem more favorable than those previously reported (RSD% ranging from 5.6 to 27.0%, reported as CV% in ref 30), although a direct comparison is not reliable due to the different analyts considered. Consistent with earlier interlaboratory studies,^30,32–34^ these results confirm that, when expressed as normalized integral ratios (*I*_anl_/*I*_ref_), NMR data acquired on different spectrometers remain directly comparable despite instrumental heterogeneity.

In the absence of established acceptance criteria specifically for NMR signal ratios, the concentration of betaine was calculated and evaluated using the Horwitz criteria,^35^ which provide an empirical benchmark for acceptable interlaboratory precision as a function of analyte concentration. Once the consensus value of *I*_bet_/*I*_TSP‑*d*4_ was determined for each sample, it was introduced into eq 9 to determine the corresponding concentration of betaine in the pasta and wheat samples. The concentrations were calculated using the data in the column “TOPSPIN” under single operator results in Table 2. This data set was selected exclusively for reasons of practical data handling convenience and does not reflect a preferential analytical choice. For each spectrum, the betaine concentration was determined individually, and the same outliers previously identified by Cochran and Huber tests were excluded prior to calculating final concentration statistics. Results of betaine quantification are reported in Table 3 together with parameters used to assess interlaboratory precision. For each sample, the table reports: (i) betaine concentration [Betaine] in millimolar units referred to the stock solution, (ii) the corresponding mass fraction *C* (*g*_betaine_/*g*_solution_), (iii) the experimental relative standard deviation (RSD%), (iv) the predicted RSD% (PRSD%) calculated according to the Horwitz equation PRSD = 2·C^–0.15^, and (v) the Horwitz ratio (HorRat), defined as the ratio between the observed and predicted RSD (HorRat = RSD%/PRSD%). The HorRat index was used as an objective measure of agreement between the experimental variability and expected analytical performance.

**Table 3:** Betaine Concentration Determined by the Internal Standard Method (Using eq 9) for Aqueous Extracts of Pasta and Wheat

	[betaine], mM	*C*, *g*_betaine_/*g*_solution_	RSD%	PRSD%	HorRat
Tube A	1.18	1.34·10^–4^	10.00	7.65	1.31
Tube B	2.01	2.28·10^–4^	6.21	7.06	0.88
Tube E	2.97	3.39·10^–4^	5.30	6.66	0.80
Tube F	2.33	2.67·10^–4^	5.40	6.90	0.78

Results reported in Table 3 highlight a high level of precision in the quantification of betaine across all analyzed samples. For the concentration values observed in this study, the PRSD% lies between 6.66 and 7.65. The experimental RSD% values fell between 5.30 and 10.00, corresponding to Horwitz ratios (HorRat) between 0.78 and 1.31. All values fall within the empirically accepted range (<2.0),^35^ indicating that the interlaboratory precision achieved is consistent with expected analytical performance at the given concentration levels.

Notably, despite potential concerns regarding the chemical behavior of TSP-*d*_4_ discussed earlier, the method demonstrates reproducibility that meets or, in several cases, exceeds typical expectations. HorRat values below 1.0 for tubes B, E, and F suggest precision better than predicted by the Horwitz model,^36,37^ while the value obtained for tube A remains comfortably within acceptable limits. These findings support the robustness of the centralized processing strategy and its suitability for collaborative quantification exercises. Overall, the results indicate that an acquisition and processing approach originally developed for nontargeted protocols, when combined with the experimentally determined correction factor α, can achieve interlaboratory precision compatible with quantitative applications, even when employing TSP-*d*_4_ as the internal standard. At the same time, the systematic behavior observed for TSP-*d*_4_ indicates that precision should be interpreted together with bias when the suitability of a quantitative reference is assessed.

### Determination of Betaine in Aqueous Extracts of Wheat and Pasta by Using External Standards: Betaine, DMSO_2_, and TSP-*d*_4_

As a complementary assessment to the internal standard approach, external standard quantification was performed to experimentally evaluate the applicability of the correction factor α introduced in the previous sections. External standard quantification was conducted by a single operator using the ERETIC2 module implemented in TOPSPIN. Despite its denomination, ERETIC2 does not refer to ERETIC (Electronic REference To access In vivo Concentration) method, but it refers to PULCON (Pulse Length Based Concentration Determination)^6,7,38^ method that is based on the use of a real reference compound measured in a separate standard solution. Quantification is achieved by comparing the analyte signal integral in the unknown sample to that of a reference compound under identical experimental conditions. Thus, the integral of the betaine peak in tubes A-F was compared against the integral of the selected peak (betaine, DMSO_2_, or TSP-*d*_4_) measured in one of the QR tubes and used by the ERETIC2 module as the external reference.

The quantification workflow began with a validation step using the reference sample QR to assess both accuracy and precision. Specifically, the betaine concentration determined by the ERETIC2 tool was compared with the gravimetrically established concentration of 1.64 mM in the stock solution, calculated from the weighed amount of betaine used in sample preparation. The results for the QR tube (Table 4) clearly illustrate the performance of the three evaluated reference compounds. When betaine itself was used as the external reference, the calculated concentration was in excellent agreement with the gravimetric value, with an exceptionally low RSD% of 0.23%, indicating excellent internal consistency under the matched conditions. Using DMSO_2_ as the external reference also yielded results in close agreement with the expected value (1.67 mM, + 1.8% deviation), with an RSD% of 0.88%, demonstrating satisfactory accuracy and precision. In contrast, when TSP-*d*_4_ was employed as the reference compound, the betaine concentration was overestimated (mean value of 1.85 mM, approximately +13% deviation). This systematic bias is consistent with observations reported in the previous sections and suggests that TSP-*d*_4_ may be affected by matrix- or environment-dependent factors, influencing its signal response. The corresponding RSD% (4.18%) was also higher than that observed with the other reference compounds, further supporting the conclusion that TSP-*d*_4_ is less reliable as an external quantitative reference under these conditions.

**Table 4:** Betaine Concentration (mM) Determined by the External Standard Method for Aqueous Extracts of Pasta and Wheat Grains

	tube QR	tube A	tube B	tube C	tube D	tube E	tube F
[betaine] by TSP-*d*_4_	1.85	1.13	2.00	2.00	3.06	3.06	2.43
RSD%	4.18	3.29	3.38	3.86	3.88	4.64	5.08
PRSD%	7.14	7.70	7.06	7.06	6.63	6.63	6.86
HorRat	0.59	0.43	0.49	0.55	0.59	0.70	0.74
Number of spectrometers participating in the consensus value	40	32	39	38	36	39	35
Outliers by Cochran test	4	11	3	6	8	5	10
Outlier by Huber test	1	2	3	1	1	1	0
[betaine] by betaine	1.64	1.01	1.77	1.77	2.70	2.70	2.14
RSD%	0.23	1.73	1.77	1.54	1.22	1.22	0.85
PRSD%	7.27	7.83	7.20	7.20	6.75	6.75	6.99
HorRat	0.03	0.22	0.25	0.21	0.18	0.18	0.12
Number of spectrometers participating in the consensus value	37	32	40	36	32	37	29
Outliers by Cochran test	6	11	3	5	9	4	11
Outlier by Huber test	2	2	2	4	4	4	5
[betaine] by DMSO_2_	1.67	1.02	1.80	1.80	2.74	2.74	2.17
RSD%	0.88	1.60	1.47	1.89	1.57	1.80	1.46
PRSD%	7.25	7.82	7.18	7.18	6.74	6.74	6.98
HorRat	0.12	0.20	0.20	0.26	0.23	0.27	0.21
Number of spectrometers participating in the consensus value	35	33	38	37	33	38	31
Outliers by Cochran test	5	10	3	5	9	4	10
Outlier by Huber test	5	2	4	3	3	3	4

Importantly, the number of laboratories identified as outliers remained limited across all three reference approaches, indicating that the observed deviations are not attributable to widespread interlaboratory inconsistencies but rather reflect the intrinsic behavior of the reference compound under the applied acquisition and processing conditions.

Only after confirming that the experimental concentrations for the QR tube fell within the acceptable uncertainty, the betaine concentration was determined in the remaining samples (tubes A–F). As summarized in Table 4, regardless of the reference compound adopted, the relative quantification of betaine follows a consistent pattern across the analyzed samples. Betaine concentrations are lower in pasta extracts (tubes A and B) and higher in wheat extracts (tubes E and F), while the paired tubes prepared without TSP-*d*_4_ (C and D) reproduced the same values as their TSP-*d*_4_-containing counterparts (B and E, respectively). It can be observed that *cv. Marco Aurelio* (B and D) is characterized by a higher content of betaine with respect to *cv. Iride* (A and F) in both pasta and wheat. In numerical terms, the results confirm the trends already observed for the QR reference sample. When TSP-*d*_4_ was used as the external reference compound, the RSD% ranged from 3.29 to 5.08. In contrast, RSD% values obtained with betaine or DMSO_2_ were less scattered, ranging from 0.88 to 1.89. The number of laboratories excluded as outliers is modest for all six tubes and, importantly, did not alter the consensus concentration values. The HorRat statistics demonstrate that the interlaboratory precision achieved with the external standard protocol exceeds the requirements predicted by the Horwitz model. When TSP-*d*_4_ was used as an external reference compound, HorRat values ranged from 0.43 (tube A) to 0.74 (tube F), significantly lower than 2.0. Employing either betaine or DMSO_2_ as the reference compound narrowed this dispersion even further. Self-referencing to betaine drove the HorRat down to 0.03 for the QR tube and 0.25 for tube B, while calibration against DMSO_2_ yielded similarly low ratios varying between 0.12 and 0.27. In every matrix examined, HorRat remains below 1, indicating reproducibility consistently better than the generic interlaboratory precision expected at these concentration levels. Although the slightly higher values obtained with TSP-*d*_4_ are very satisfactory within the acceptable limits, they corroborate earlier observations that this signal is more susceptible to subtle matrix-dependent variability, whereas betaine and DMSO_2_ provide markedly more robust reference compounds for quantitative calibration across laboratories.

## Conclusions

The results of this interlaboratory comparison validate the central premise of the study: nontargeted NMR analysis, when anchored to gravimetrically traceable calibration standards by a correction factor α (eq 8), can achieve a reproducibility comparable to that of dedicated quantitative approaches. Under nonideal conditions, the correction factor α provides a protocol-dependent linkage between spectroscopic integral ratios and gravimetrically traceable molar ratios, enabling reproducible quantification by internal standard methods in the absence of calibration curves.

As a case study, the betaine concentration was determined in aqueous extracts of wheat or pasta in the framework of an interlaboratory comparison involving 50 laboratories that recorded a total of 1750 NMR spectra. It was demonstrated that the introduction of the correction factor α led to satisfactory reproducibility when TSP-*d*_4_ was used as an internal standard. External standard quantification validated the applicability of the correction factor α and demonstrated that NMR spectra acquired for screening purposes can be used to quantify analytes contained in complex mixtures. Accuracy and precision of the external calibration were evaluated with three reference compounds, namely, betaine, DMSO_2_, and TSP-*d*_4_. Using the betaine as a self-reference reproduced the gravimetric concentration of 1.64 mM exactly and afforded HorRat values far better than the generic interlaboratory precision predicted by the Horwitz function. Similarly, using DMSO_2_ as a reference, low HorRat values were obtained with a positive bias of about 2%. In contrast, the use of TSP-*d*_4_ as a reference led to a systematic overestimation (>10%) of betaine concentration and higher standard deviations, even though the corresponding HorRat values remained under the accepted threshold (<2.0).

Collectively, these observations show that in the practical context of cereal-matrix extracts, betaine and DMSO_2_ provide both accurate and highly precise results by external standard quantification, while TSP-*d*_4_ systematically overestimates the betaine concentration in both external and internal standard quantifications. Consequently, TSP-*d*_4_ should be used only as a chemical-shift reference.

It is important to consider that all spectra were processed both locally (multioperator) and centrally (single operator). The comparison of these two data sets shows unambiguously that the largest source of variability does not arise from differences in hardware or field strength but from the way spectra are processed. Based on the data presented, it is evident that the software used for processing raw NMR data has a negligible effect on the determination of the calculated betaine concentrations.

Results validate the present approach as a robust tool for routine food-quality control and metabolite profiling. A key advantage of this methodology is that a single 1D ^1^H NOESY experiment, originally optimized for nontargeted metabolite profiling, can simultaneously provide high-throughput profiling data and serve as a basis for metrologically sound quantitative determination of selected metabolites. This dual functionality is particularly advantageous for routine laboratories, as it minimizes method proliferation and enables quantitative exploitation of existing profiling data without the need for additional dedicated qNMR acquisitions. In this way, the role of nontargeted methodologies can be redefined, positioning them not merely as preliminary tools but as reliable alternatives capable of supporting decision-making in contexts demanding both efficiency and analytical rigor.

An additional advantage of these findings consists of the opportunity to quantify selected analytes contained in complex mixtures that have already been analyzed in previous studies adopting an analytical NMR protocol optimized for screening purposes (sample preparation, choice of the internal standard, definition of acquisition, and processing parameters). For example, if a metabolomics screening reveals the presence of analytes of interest, the already recorded NMR spectra can be used for rigorous *a posteriori* quantification. This implies the calculation of the correction factor α, which requires recording the NMR spectra of a suitable reference mixture containing the same internal standard and maintaining the same acquisition and processing parameters adopted in the optimized protocol.

## Supplementary Material



## Notes

List of the NMR tubes submitted to ILC with the corresponding sample composition, list of some acquisition parameters depending on the magnetic field of the spectrometer in use, stability tests, and *T*_1_ determinations for tubes QR, A, and F (PDF)

## †††††

INRAE, CALIS/PROBE Research Infrastructures, BIBS facility, F-44000 Nantes, France

## ‡‡‡‡‡

INRAE, UR BIA, F-44000 Nantes, France

## Notes

This work is dedicated to Maurizio Triggiani, a key contributor to the design and realization of this study, who sadly passed away before its publication. He is remembered with gratitude and esteem.
