# Supplementary material for: Harmonized 1H NMR Workflow Enables Quantitative Betaine Determination from Nontargeted Metabolite Profiling Using Internal and External Standards
Source: Anal Chem. 2026 May 29;98(22):15995–6007. doi: 10.1021/acs.analchem.5c05198 (PMC13261621; doi:10.1021/acs.analchem.5c05198)
Supplement: Supplementary file 1 [file ac5c05198_si_001.pdf]

Harmonized ^{1}H NMR Workflow Enables Quantitative Betaine Determination from Non-Targeted Metabolite Profiling Using Internal and External Standards

Biagia Musio[a], Maria Trisolini[a], Rosa Ragone[a], Stefano Todisco[a], Antonino Rizzuti[a], Piero Mastrorilli[a,b], Mario Latronico[a,b], Nicola Intini[b,c], Annamaria Greco[a], Marica Antonicelli[a], Cristina Airoldi[d], Luca Antoniacomi[e], Luca Goldoni[f], Mihaela Balan-Porcărașu[g], Francesca Benevelli[h], Davide Bertelli[i], Aurimas Bieliauskas[j], Luana Bontempo[k], Asma Bourafai-Aziez[l], Diego Brancaccio[m], Emanuela Callone[n], Angeles Canales Mayordomo[o], Enrico Caneva[e], Greta Petrella[p], Roberto Consonni[q], Iain J. Day[r], Catherine Deborde[s,#,£], Calin Deleanu[t], Giacomo Di Matteo[u], Cătălin Duduianu[v], John Edwards[w], Luca Fusaro[x], Sylvie Gehanne[y], Nicola Genna[z], Dessislava Gerginova[aa], Roberto Gobetto[ab], Gonzalo Hernandez[ac], Nunzia Iaccarino[m], Pasquale Illiano[e], István Timári[ad], Thomas Kuballa,[ae] Dirk W. Lachenmeier,[ae] Yavor Mitrev[aa], Roland Molinie[af], Adele Mucci[ag], Claudia Napoli[h], Alina Nicolescu[g], Valentina Petrelli[ah], Luca Piemontese[ai], Chiara Portesi[aj], Antonio Randazzo[m], Teresa Recca[ak], Algirdas Šačkus[j], Mirjam Schmidt[al], Svetlana Simova[aa], Anatoly Petrovich Sobolev[am], Pavel Solovyev[k], Mattia Spano[u], Jan C. Teipel[ae], Marina Veronesi[an], Vito Gallo*[,a,b]

a) Dipartimento di Ingegneria Civile, Ambientale, del Territorio, Edile e di Chimica (DICATECh), Politecnico di Bari, Via Orabona 4, I-70125, Bari, Italy.

b) Innovative Solutions, Innovative Solutions S.r.l, Spin Off del Politecnico di Bari, Zona H 150/B, I-70015, Noci (BA), Italy.

c) Agenzia Regionale per la Prevenzione e la Protezione dell'Ambiente, ARPA Puglia, Corso Trieste 127, I-70126, Bari, Italy.

d) Dipartimento di Biotecnologie e Bioscienze, Università of Milano-Bicocca, P.zza della Scienza 2, I-20126, Milano, Italy.

e) Comprehensive Substances characterization via advanced Spectroscopy (COSPECT), Università degli studi di Milano, Via Golgi 19, I-20133 Milano, Italy.

f) Analytical Chemistry Facility, Fondazione Istituto Italiano di Tecnologia (IIT), Via Morego 30, Genoa, I-16163 Italy.

g) "Petru Poni" Institute of Macromolecular Chemistry, 41A Gr. Ghica Voda Alley, Iasi, 700487, Romania

h) Bruker Italia S.r.l., Viale V. Lancetti 43, I-20158, Milano, Italy.

i) Dipartimento Scienze della Vita, Università di Modena e Reggio Emilia, Via campi 103, I-41125, Modena, Italy.

j) Institute of Synthetic Chemistry, Kaunas University of Technology, K. Baršausko Str. 59, LT-51423, Kaunas, Lithuania.
k) Traceability Unit, Research and Innovation Center – Fondazione Edmund Mach (F.E.M), Via E. Mach 1, 38098, San Michele all'Adige (TN), Italy.
l) EVEAR EXTRACTION, 48 Route de Gennes, LD Félines, CEDEX 4, F-49320 Angers, France.
m) Dipartimento di Farmacia, Università di Napoli Federico II, Via D. Montesano 49; Napoli, Italy.
n) Dipartimento di Ingegneria Industriale, Università di Trento, Via Sommarive 9, I-38123, Trento, Italy.
o) Departamento Química Orgánica I, Facultad Ciencías Químicas, Universidad Complutense de Madrid, Avenida Complutense s/n., S-28040, Madrid, Spain.
p) Dipartimento di Scienze e Tecnologie Chimiche, Università di Roma "Tor Vergata", I-00133, Roma, Italy.
q) Istituto di Scienze e Tecnologie Chimiche "G. Natta" (SCITEC), Consiglio Nazionale delle Ricerche (CNR), Via Corti 12, Milan, I-20133, Italy.
r) JEOL UK Ltd, 4 Bankside, Long Hanborough, Oxfordshire, OX29 8LJ, U.K..
s) MetaboHUB-Bordeaux, Centre INRAE de Nouvelle-Aquitaine Bordeaux, 71 Avenue E. Bourlaux, F-33140 Villenave d'Ornon, France.
t) "C.D. Neniţescu" Institute of Organic and Supramolecular Chemistry, 202-B Spl. Independenţei, Bucharest, RO-060023, Romania.
u) Dipartimento di Chimica e Tecnologie del Farmaco, Sapienza Università di Roma, Piazzale Aldo Moro 5, I-00185, Roma, Italia.
v) Faculty of Chemical Engineering and Biotechnologies, National University for Science and Technology Politehnica Bucharest, Bucharest, RO-011061, Romania.
w) Process NMR Associates, LLC, 84 Patrick Lane, Suite 115, Poughkeepsie, NY 12603, United State.
x) Chemistry Department; University of Namur, rue de Bruxelles 61, Namur, 5000, Belgium.
y) Aptuit S.r.l. an Evotec company, Via Fleming 4, Verona, 37135, Italy.
z) Lab. Instruments S.r.l., SS172 Putignano-Alberobello Km 28+200, Castellana Grotte (BA), 70013, Italy.
aa) Institute of Organic Chemistry with Centre of Phytochemistry; Bulgarian Academy of Sciences, Akad. G. Bonchev bl. 9, Sofia, 1113, Bulgaria.
ab) Dipartimento di Chimica, Università di Torino, Via P. Giuria, 7, Torino, I-10125, Italy.
ac) Laboratorio de Resonancia Magnética Nuclear, Departamento de Química Orgánica, Facultad de Química, Universidad de la República, Montevideo, 11800, Uruguay.
ad) Department of Organic Chemistry, Faculty of Science and Technology, University of Debrecen, Egyetem tér 1, Debrecen, H-4032, Hungary
ae) Chemisches und Veterinäruntersuchungsamt (CVUA) Karlsruhe, Weissenburger Strasse 3, Karlsruhe, 76187, Germany.

af) Transfrontalière BioEcoAgro, INRE 1158 Biologie des Plantes et Innovation (BIOPI), UPJV, 1; Rue des Louvels, Amiens, 80037, France.
ag) Dipartimento di Scienze Chimiche e Geologiche, Università di Modena e Reggio Emilia, Via G. Campi 103, 41125, Modena, Italy.
ah) FONDAZIONE ITS Istituto Tecnico Superiore Area “Nuove Tecnologie per il Made in Italy”, Settore produzioni agroalimentari; S.C. 138 C.da Marangi n.26, 70010 Locorotondo (Ba), Italy.
ai) University of Bari Aldo Moro, Dipartimento di Farmacia-Scienze del Farmaco, campus E. Quagliariello - Palazzo V. Tortorella, via E. Orabona 4, 70126 Bari, Italy
aj) INRIM Istituto Nazionale di Ricerca Metrologica, Strada delle cacce 91, Torino, 10135, Italy.
ak) Centro Grandi Strumenti, Università di Pavia, Via Bassi 21, Pavia, 27100, Italy.
al) Lower Saxony State Office for Consumer Protection and Food Safety (LAVES); Food and Veterinary Institute Braunschweig/Hannover, Dresdenstrasse 2, Braunschweig, 38124, Germany.
am) Istituto per i Sistemi Biologici; Consiglio Nazionale delle Ricerche CNR; via Salaria km 29.300, Monterotondo (RM), 00015, Italy.
an) Structural Biophysics Facility, Fondazione Istituto Italiano di Tecnologia (IIT), Via Morego 30, Genoa, I-16163, Italy

Present address:
#) INRAE, CALIS/PROBE Research Infrastructures, BIBS facility, F-44000 Nantes, France
£) INRAE, UR BIA, F-44000 Nantes, France

Harmonized ^{1}H NMR Workflow Enables Quantitative Betaine Determination from Non-Targeted Metabolite Profiling Using Internal and External Standards 1
Table S1. List of the NMR tubes submitted to ILC with the corresponding sample composition .. 5
Table S2. List of some acquisition parameters depending on the magnetic field of the spectrometer in use 5
Stability test 5
T_1 Determination on Tube QR at 400 MHz and 298 K 9
T_1 Determination on Tube A (pasta) at 400 MHz and 298 K 11
T_1 Determination on Tube F (wheat) at 400 MHz and 298 K – plot M_0 *vs* t 12

Table S1. List of the NMR tubes submitted to ILC with the corresponding sample composition

Tube label	Solutions
T	600 µL of Methanol-d_4
QR	630 µL of aqueous solution of reference compounds ([betaine] = 1.64 mM, [$DMSO_2$] = 2.10 mM, [TSP-d_4] = 1.12 mM) + 70 µL of D_2O
A	630 µL of aqueous extracts of pasta (cv. *Iride*) + 70 µL of TSP-d_4 in D_2O (0.20 %, w/w)
B	630 µL of aqueous extracts of pasta (cv. *Marco Aurelio*) + 70 µL of TSP-d_4 in D_2O (0.20 %, w/w)
C	630 µL of aqueous extracts of pasta (cv. *Marco Aurelio*) + 70 µL of D_2O
D	630 µL of aqueous extracts of durum wheat (cv. *Marco Aurelio*) + 70 µL of D_2O
E	630 µL of aqueous extracts of durum wheat (cv. *Marco Aurelio*) + 70 µL of TSP-d_4 in D_2O (0.20 %, w/w)
F	630 µL of aqueous extracts of durum wheat (cv. *Iride*) + 70 µL of TSP-d_4 in D_2O (0.20 %, w/w)

Table S2. List of some acquisition parameters depending on the magnetic field of the spectrometer in use

	Magnetic Field (MHz)					
	**80**	**300**	**400**	**500**	**600**	**700**
**Time Domain (points)**	10240	38400	51200	64000	76800	89600
**Spectral width (Hz)**	1200	4500	6000	7500	9000	10500
**Dwell time (µs)**	416.7	111.1	83.3	66.7	55.6	47.6

Stability test

The lack of statistical data from interlaboratory comparisons, and in particular of reference standard deviations for proficiency assessment, prevented the execution of homogeneity test according to typical procedures. The tests for the so-called "sufficient homogeneity" are aimed to ascertain, before sample delivery, the negligibility of the variation in composition with respect to variation introduced

by the measurements carried out by participants. In the present ILC, the only way to evaluate the sufficient homogeneity of the samples was to compare, after data acquisition, the variation in composition with the variation introduced by the measurements carried out by participants.

According to technical requirements of ISO 17043:2010, the homogeneity of the samples and the stability of the investigated matrices was checked. This procedure is aimed to ascertain, before sample delivery, the negligibility of the variation in composition with respect to variation introduced by the measurements conducted by participants.

An NMR tube prepared from the bulk solution of durum wheat was sealed and left at room temperature. 1D ^{1}H NOESY spectra were recorded after preparation (0 h) and after 48 h, 4 days, 6 days, and 13 days (see spectra in Figure S1). The stability of the sample was assessed by integrating the areas of three signals:

- S1, the doublet of alanine (1.48 ppm);
- S2, the singlet of betaine (3.25 ppm);
- S3, a doublet belonging to the tryptophan signals pattern (7.52 ppm).

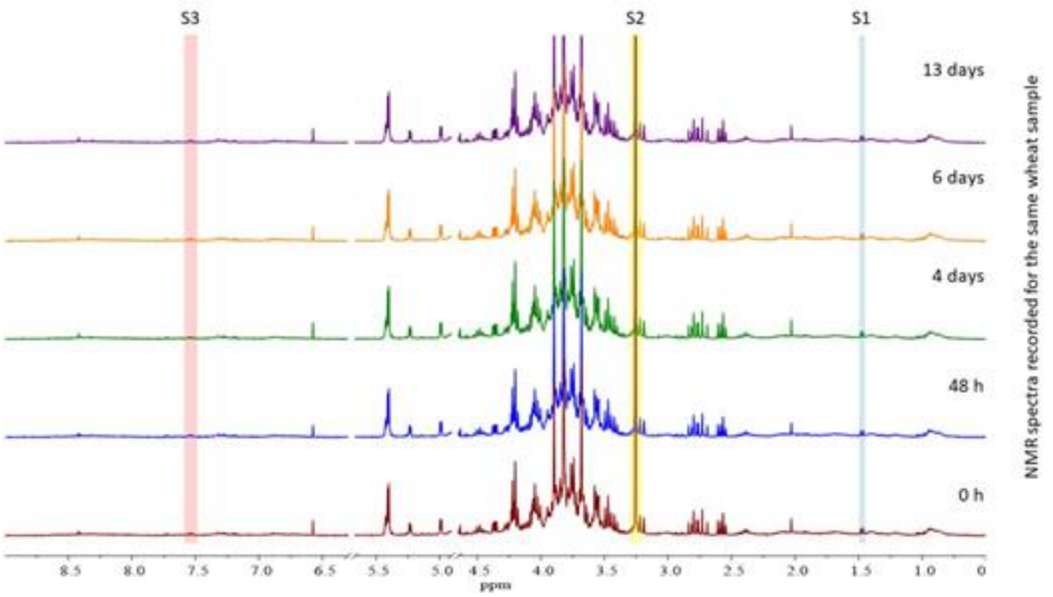

Figure S1. 1D ^{1}H NOESY spectra (400 MHz) of the durum wheat sample used for the stability test.

For signal S1, the spectral area considered for the analysis of the integrals was in the range [1.45÷1.50 ppm]; for signal S2, the integration range was [3.24÷3.26 ppm]; for signal S3, the integration range was [7.51÷7.55 ppm]. The integrals were expressed in absolute values. Then, the following statistical parameters were calculated: mean (μ), standard deviation (σ) and, the coefficient of variation percentage (CV%). The data obtained from those calculations are collected in Table 1.

Table S3. Results of the stability test of durum wheat sample

	S1	S2	S3
**Integration range (ppm)**	[1.50÷1.45]	[3.26÷3.24]	[7.55÷7.51]
**Time**		**Integral values**	
**0 h**	873.7	17264.0	315.6
**48 h**	845.6	16159.6	325.3
**4 days**	926.2	16959.6	342.8
**6 days**	911.6	16989.5	338.5
**13 days**	875.9	17070.3	330.4
**Average**	886.6	1688.6	330.5
**Dev std**	32.2	424.4	10.8
**CV%**	3.6	2.5	3.3

The calculated CV% values for the three selected signals were below 5% (3.6 for S1, 2.5 for S2, and 3.3 for S3).

Moreover, the stability of the pasta samples over two weeks was evaluated. An NMR tube prepared from the bulk solution of pasta was sealed and left at room temperature, to record 1D ^{1}H NOESY spectra after 0, 3, 7, and 14 days (Figure S2).

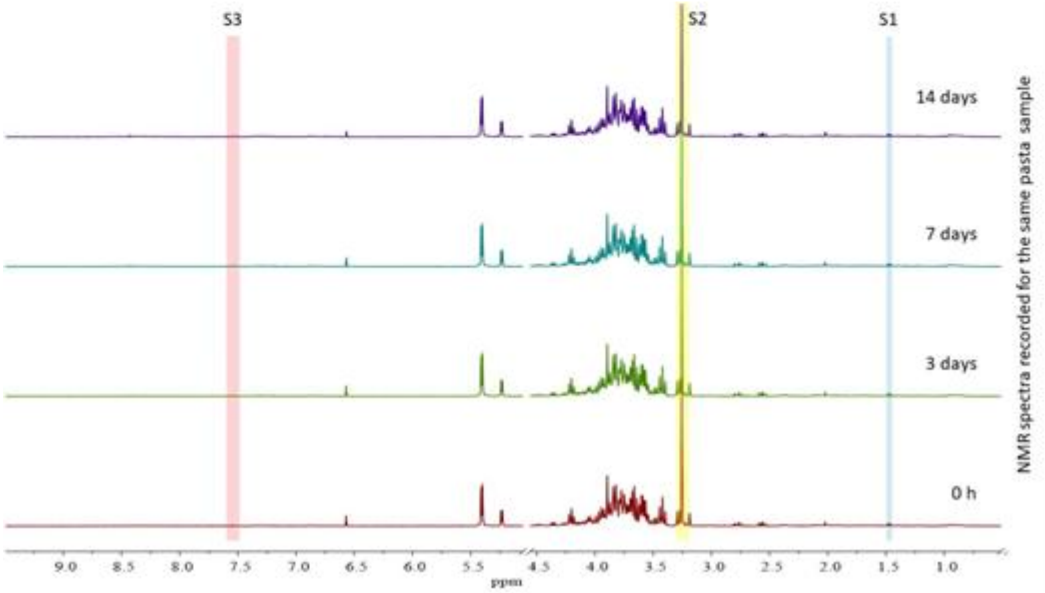

Figure S2. 1D ^{1}H NOESY spectra (400 MHz) of the pasta sample used for the stability test.

For the evaluation of the stability of the pasta sample, the same signals selected for wheat stability were considered (S1, S2, and S3).

The results of the stability test of pasta are collected in Table S2.

Table S4. Results of the stability test of pasta sample

	**S1**	**S2**	**S3**
**Integration range (ppm)**	[1.50÷1.45]	[3.26÷3.24]	[7.55÷7.51]
**Time**		**Integral values**	
**0 h**	541.7	11408.7	215.9
**3 days**	550.2	11831.6	217.3
**7 days**	592.16	11855.4	193.9
**14 days**	627.9	12394.4	193.7
**Average**	577.9	11872.5	205.2
**Dev std**	39.9	403.9	13.2
**CV%**	6.9	3.4	6.4

The CV% values calculated for signals S1 and S3 resulted in being higher than 5%. On the other hand, the CV% of signal S2 of betaine was below 5%.

T_1 Determination on Tube QR at 400 MHz and 298 K

$$M = M_0(1 - 2 \cdot e^{-t/T1})$$

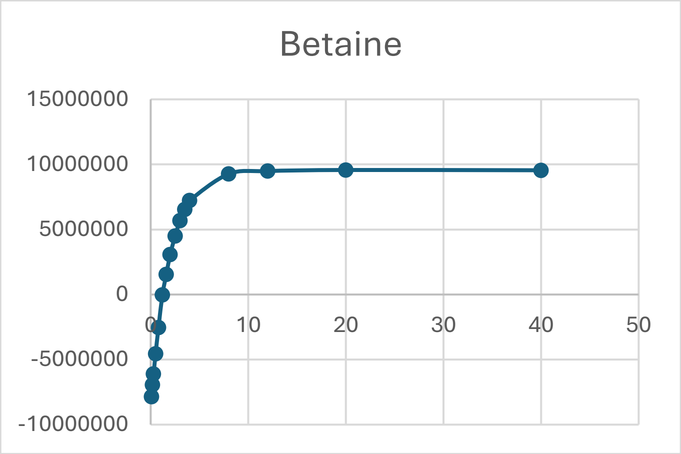

Figure S3. Plot M_0 *vs* t for betaine

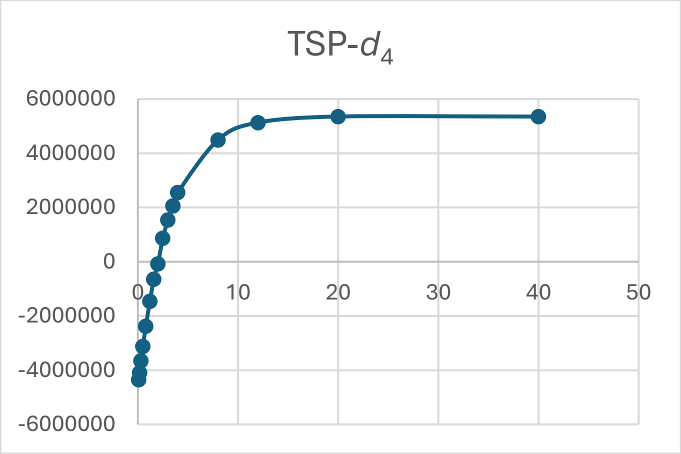

Figure S4. Plot M_0 *vs* t for TSP-d_4

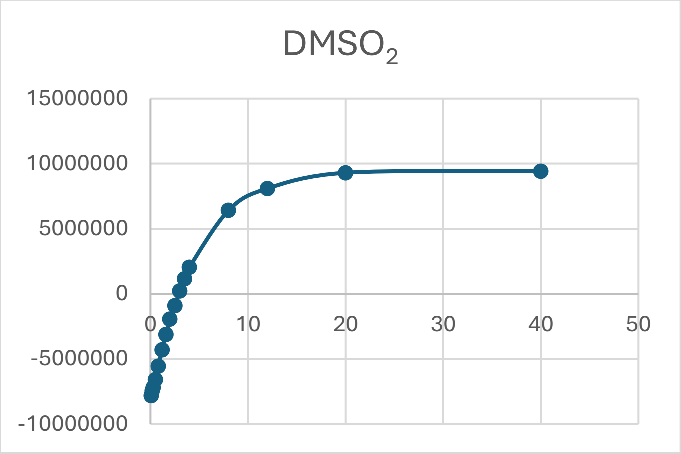

Figure S5. Plot M_0 *vs* t for $DMSO_2$

$$\ln(M_0 - M) = \ln(2 \cdot M_0) - \frac{t}{T_1}$$

Table S5. T_1 values calculated for betaine, TSP-*d*$_4$ and $DMSO_2$ by plotting $\ln(M_0$-$M)$ *vs* t

Compound	$\frac{1}{T_1}$	$\ln(2 \cdot M_0)$	R^2	T_1 (s)
Betaine	0.49	16.67	0.9972	2.05
TSP-*d*$_4$	0.31	16.11	0.9995	3.18
$DMSO_2$	0.24	16.74	0.9952	4.17

T_1 Determination on Tube A (pasta) at 400 MHz and 298 K

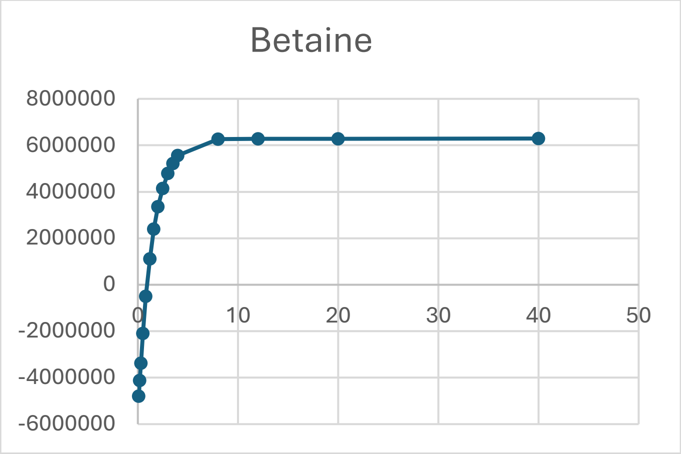

Figure S6. Plot M_0 *vs* t for betaine

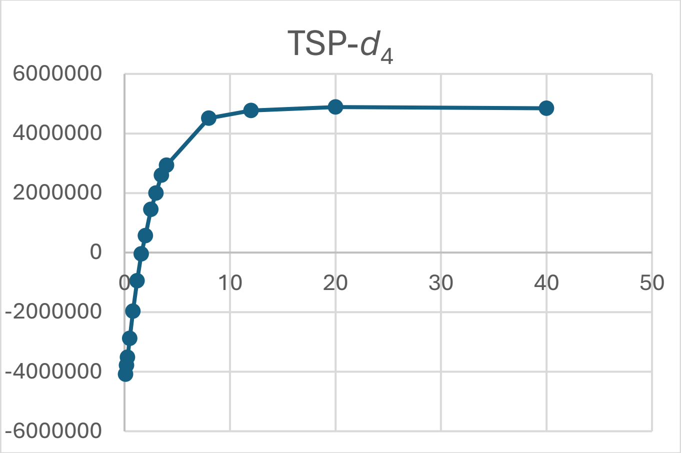

Figure S7. Plot M_0 *vs* t for TSP-d_4

$$\ln(M_0 - M) = \ln(2 \cdot M_0) - \frac{t}{T_1}$$

Table S6. T_1 values calculated for betaine and TSP-d_4 by plotting ln(M_0-M) *vs* t

**Compound**	$-\frac{1}{T_1}$	$\ln(2 \cdot M_0)$	**R^2**	**T_1 (s)**
Betaine	-0.73	16.35	0.9982	1.36
TSP-d_4	-0.41	16.05	0.9995	2.45

T_1 Determination on Tube F (wheat) at 400 MHz and 298 K – plot M_0 *vs* t

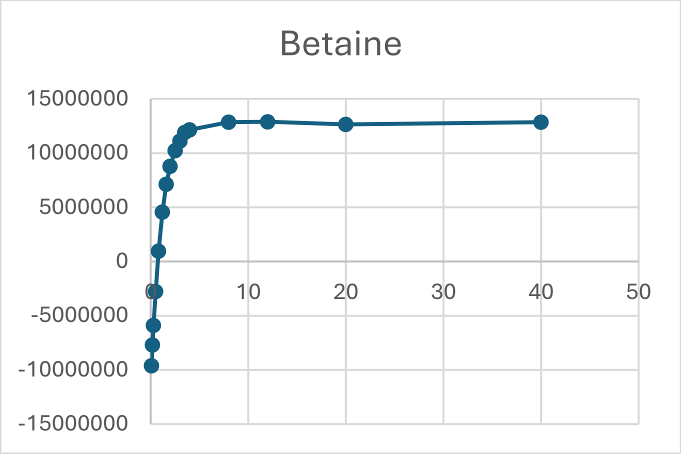

Figure S8. Plot M_0 *vs* t for betaine

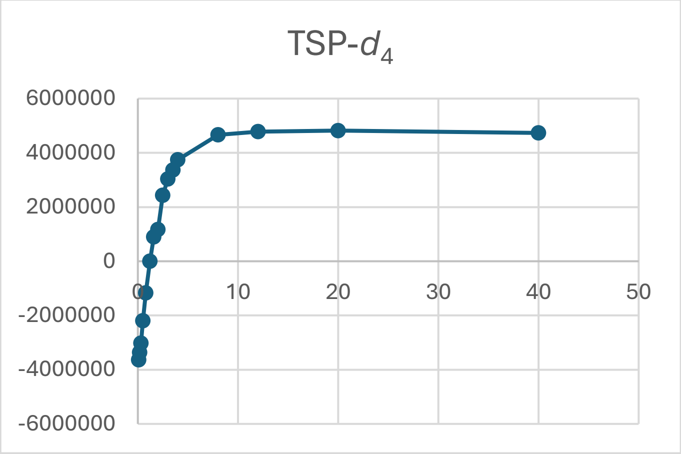

Figure S9. Plot M_0 *vs* t for TSP-d_4

$$\ln(M_0 - M) = \ln(2 \cdot M_0) - \frac{t}{T_1}$$

Table S7. T_1 values calculated for betaine and TSP-d_4 by plotting ln(M_0-M) *vs* t

Compound	$-\frac{1}{T_1}$	$\ln(2 \cdot M_0)$	R^2	T_1 (s)
Betaine	-0.90	17.02	0.999	1.10
TSP-d_4	-0.60	16.11	0.9943	1.66
